# Controlling hypothalamic DNA methylation at the Pomc promoter does not regulate weight gain during the development of obesity

**DOI:** 10.1371/journal.pone.0284286

**Published:** 2023-04-10

**Authors:** Taylor McFadden, Natasha Gaito, Isabella Carucci, Everett Fletchall, Kayla Farrell, Timothy J. Jarome

**Affiliations:** 1 School of Animal Sciences, Virginia Polytechnic Institute and State University, Blacksburg, VA, United States of America; 2 School of Neuroscience, Virginia Polytechnic Institute and State University, Blacksburg, VA, United States of America; Indiana University Purdue University at Indianapolis, UNITED STATES

## Abstract

Obesity is a complex medical condition that is linked to various health complications such as infertility, stroke, and osteoarthritis. Understanding the neurobiology of obesity is crucial for responding to the etiology of this disease. The hypothalamus coordinates many integral activities such as hormone regulation and feed intake and numerous studies have observed altered hypothalamic gene regulation in obesity models. Previously, it was reported that the promoter region of the satiety gene, *Pomc*, has increased DNA methylation in the hypothalamus following short-term exposure to a high fat diet, suggesting that epigenetic-mediated repression of hypothalamic *Pomc* might contribute to the development of obesity. However, due to technical limitations, this has never been directly tested. Here, we used the CRISPR-dCas9-TET1 and dCas9-DNMT3a systems to test the role of *Pomc* DNA methylation in the hypothalamus in abnormal weight gain following acute exposure to a high fat diet in male rats. We found that exposure to a high fat diet increases *Pomc* DNA methylation and reduces gene expression in the hypothalamus. Despite this, we found that CRISPR-dCas9-TET1-mediated demethylation of *Pomc* was not sufficient to prevent abnormal weight gain following exposure to a high fat diet. Furthermore, CRISPR-dCas9-DNMT3a-mediated methylation of *Pomc* did not alter weight gain following exposure to standard or high fat diets. Collectively, these results suggest that high fat diet induced changes in *Pomc* DNA methylation are a consequence of, but do not directly contribute to, abnormal weight gain during the development of obesity.

## Introduction

Within the U.S. population obesity has reached epidemic proportions and shows no signs of slowing as new studies predict that by the year 2030 nearly 1 in 2 adults will be obese [1, 2]. Obesity, which is defined as excess body fat concentration and storage of adipocytes within the body, results in an annual cost of more than $340 billion in health care and is a common risk factor for many diseases, such as diabetes and cancer [3–5]. As a result, understanding the neurobiology of obesity is crucial for responding to the etiology of this disease.

The hypothalamus is responsible for controlling both food intake and energy expenditure in order to maintain the body’s energy balance. It has been shown that neuroinflammation is increased and gene expression is altered in the hypothalamus during the development of obesity [6–8]. Epigenetic mechanisms can lead to persistent changes in transcription and could be an important mechanism controlling altered gene regulation in several brain regions, including the hypothalamus, in rodent diet induced obesity (DIO) models [9–11]. Consistent with this, altered DNA methylation levels at crucial metabolic genes connected to obesity have been reported [12]. For example, studies have shown that within rodent DIO models, there are decreases in *Pomc* (Proopiomelanocortin) expression, which codes for an important appetite regulatory enzyme that gives rise to a wide range of satiety hormones and has been implicated in obesity [13], and this is related to increased DNA methylation of the *Pomc* promoter [14]. Further, *Pomc* deficiency leads to the development of obesity [15–18]. Together, these data strongly implicate a role for *Pomc* repression in the development and progression of obesity. Despite this evidence, the link between DNA methylation changes at *Pomc* in the hypothalamus and the development of obesity are not well understood as they have never been directly tested due to technical limitations that prevent the precise targeting of DNA methylation enzymes to this specific genomic region. However, the advent of the CRISPR revolution has changed this by providing highly precise dead Cas9 (dCas9) fusions with specific DNA methylation enzymes, including DNMT3a and TET1 [19, 20], which regulate transcriptionally repressive and active DNA 5-methylcytosine (5-mC) and 5-hydroxymethylcytosine (5-hmC), respectively. Despite this, these dCas9-DNA methylation fusions have rarely been reported to be used in the brain and have yet to be applied to DIO models.

Here, we used a common DIO procedure in combination with precise CRISPR-dCas9-TET1 and dCas9-DNMT3a mediated manipulations of the *Pomc* promoter to determine the contribution of *Pomc* DNA methylation changes to abnormal weight gain during the development of obesity. We found that direct manipulations of *Pomc* promoter DNA 5-mC and 5-hmC levels were unable to alter weight gain in a rat DIO model. Collectively, these results have important implications for better understanding the underlying genetic changes that lead to obesity development and the importance of DNA methylation changes of major appetite suppression genes following abnormal weight gain.

## Materials and methods

### Subjects

A total of 66 male 8–9-week-old Sprague Dawley rats were obtained from Envigo (Frederick, MA). Animals were group housed two per cage on ventilated racks with access to food and water ad libitum for 2 days before the experiment began. The colony room was maintained under a 12:12-hr light/dark cycle. Experiments took place during the light portion of the cycle. All procedures were approved by the Virginia Polytechnic Institute and State University Institutional Animal Care and Use Committee (protocol number 21–207) and conducted within the ethical guidelines of the National Institutes of Health.

### CRISPR plasmid preparation

Targeting of TET1 and DNMT3a to the *Pomc* promoter was performed using the CRISPR-dCas9 system via our previously described procedure [21, 22]. CRISPOR was used to design gRNAs targeting a 200bp region of the *Pomc* promoter. The oligo was then cloned into a sgRNA expression vector driven by the murine U6 promoter (Addgene plasmid #44248) and transformed into chemically competent E. coli. Clones were grown overnight in broth with 100mg/ml ampicillin, DNA extracted using the Qiagen MiniPrep kit and Sanger sequenced at the Virginia Tech Fralin Life Science Institute. Clones containing the correct sequence were grown in broth with 100mg/ml ampicillin and endotoxin-free plasmids were collected using the Clontech EF-MidiPrep Kit. The dCas9 was fused to the TET1 transactivator domain (plasmid #83340) or the CRISPR-dCas9-DNMT3a (plasmid #71666). On the day of surgery, endotoxin-free plasmids were then diluted to a final concentration of ~500ng/μl in sterile 10% glucose and incubated with in vivo JetPEI (Polyplus) for 15 min at room temperature.

### Cell culture

Rat B35 neuroblastoma cell line (#CRL-2754; ATCC, Manassas, VA, USA) were cultured in Dulbecco’s Modified Eagle’s Medium (DMEM) (#30–2002; ATCC, Manassa, VA, USA) supplemented with 10% Fetal Bovine serum (#35-016-CV; Corning, Tewsbury, MA, USA) and 0.1% Penicillin/Streptomycin (#15070063; Gibco, Gaitherburg, MD, USA). One day prior to transfection, cells at 70–90% confluency in a 100 mm dish were treated with 0.05% Trypsin-EDTA (1X) (#25300054; Gibco, Gaitherburg, MD, USA). Cells were then placed into a 6-well dish with a 1:6 ratio of cells going into each well containing 2ml of DMEM-based media. Transfection was conducted using Lipofectamine 3000 reagent (L3000001; Invitrogen, Carlsbad, CA) following manufacturer’s instructions. Briefly, on the day of transfection, DMEM-based media was removed and cells were washed with DPBS (#14190144; Gibco). Lipofectamine reagents and DNA plasmids were mixed following manufacturer’s instructions and 250μl of the DNA-lipid complex was combined with 1.75ml Opti-MEM media (#31985070; Gibco) in each well. In the 6-well dish, 3 wells were transfected with the gRNA and SYN-dCas9-VPR (Addgene, plasmid #114196), and 3 wells were transfected with only the gRNA. Cells were cultured in a NAPCO series 8000 Water Jacket CO2 incubator (model 3586) for 48 hours post transfection. RNA was isolated from the cells by TRIzol (#15596018; Ambion, Austin, TX) following the manufacturer’s instructions. cDNA synthesis and real-time PCR were performed as described below.

### Surgery

Animals underwent stereotaxic surgeries where CRISPR-dCas9 plasmids were injected into the dorsalmedial hypothalamus using coordinates relative to Bregma (A/P: -3.3mm, M/L: +/- 0.5mm, D/V: -8.8mm) as described previously [22]. Animals were anesthetized with 1–4% isoflurane, hypothalamus location determined, and holes drilled using standard procedures. CRISPR-dCas9 plasmids were injected bilaterally into the hypothalamus using a 26-gauge Hamilton syringe connected to an automated pump (Harvard Apparatus) at a rate of 0.1μl per minute for a total of 0.5μl per hemisphere. Animals were then given a subcutaneous injection of carprofen (10mg/kg) and topical lidocaine on the day of surgery to assist in pain relief.

### Obesity paradigm

In the first two experiments, 20 male rats (per experiment) were randomly divided into two groups with similar average body weight. For seven weeks the animals were fed one of two diets. The first group was fed a control diet that consisted of standard rat chow (22% protein, 4% fat, 5% fiber), while the second group was fed a high fat diet (60% kcal fat; #D12492, Research Diets, Inc., New Brunswick, NJ). All animals were given 120 grams of feed and body weight and food intake were measured twice per week (every 3–4 days on Tuesdays and Fridays) over the course of 6 weeks; animals remained on the diet during the seventh week but were not weighed to avoid unnecessary stress during the final week before tissue collection. On Day 49, the animals were euthanized, and brain tissue was dissected. Experiment 1 tissue was processed for DNA while Experiment 2 was for RNA. In the third experiment, 24 male rats were randomly divided into three groups (n = 8 per group): Two Control groups, infused with only the Pomc-gRNA, and an Experimental group infused with the Pomc-gRNA+dCas9-TET1 into the hypothalamus to upregulate 5-hmC at the *Pomc* promoter prior to the DIO procedure. Two weeks post infusion, 8 control animals were fed a standard rat chow diet for 7 weeks. The 8 additional control and 8 dCas9-Tet1 animals were fed a high fat diet for 7 weeks. During the first six weeks, weight and food intake was recorded. At the end of the seventh week, we euthanized the animals and collected hypothalamus tissue. In the fourth experiment, 20 male rats were randomly divided into two groups, Control (Pomc-gRNA alone, n = 12) or Pomc-gRNA+dCas9-DNMT3a (n = 8). Two weeks post infusion, all animals were fed standard rat chow diet for 6 weeks, followed by a high fat diet for 4.5 weeks, both of which we have found to be sufficient for significant changes in body weight based on the diet used. At the middle of week eleven, animals were euthanized and hypothalamus tissue collected. Every attempt was made to ensure that all personnel were blinded to the experimental conditions, albeit that diet provided was not able to be blinded due to significant differences in color and texture.

### Tissue collection

At the end of the diet procedure, animals were overdosed on isoflurane in a neocrosis chamber and rapidly decaptitated. Brains were removed, flash frozen on dry ice and stored at -80°C until dissection.

### DNA extraction

Dissected hypothalamus samples were homogenized in TE buffer with 1% SDS and 100μg of proteinase K using a Teflon homogenizer. Homogenized samples were then incubated for 2 hours at 55°C and DNA was extracted by phenol/chloroform/isoamyl alcohol and then ethanol precipitated. DNA concentration was measured on the Take3 (BioTek, Winooski, VT, USA).

### RNA extraction

Using dissected hypothalamus tissue, high quality RNA was extracted using the Qiagen Allprep Kit and stored at -80°C for later use.

### cDNA synthesis and quantitative real-time PCR

RNA concentrations were measured on the Take3 (BioTek), normalized (200 ng), and converted to cDNA using the iScript cDNA synthesis kit (Bio-rad, Hercules, CA, USA). Real-time PCR amplifications of the cDNA were performed on the Bio-rad CFX96 Real-Time System using the following protocol: 95.0°C for 3 min, then 95.0°C for 10 s, followed by 60°C for 30 s (39 repeats), 55–95°C for 0.5°C/cycle, followed by a melt curve starting at 55.0°C for 10 s (81 repeats), and then held at 4.0°C. Primers were: Pomc (F: GACCAAACGGGAGGCGACGG; R: GGCTCTGTCGCGCAAAGGCA) and Gapdh (F: ACCTTTGATGCTGGGGCTGGC; R: GGGCTGAGTTGGGATGGGGACT) used as an internal control and data was analyzed using the comparative Ct method.

### Methylated DNA immunoprecipitations (meDIPs)

DNA extracted from hypothalamus was subjected to meDIP analysis as described previously [23]. MagnaChIP protein A+G beads were washed in 0.5% BSA blocking buffer and bound overnight with primary antibody against 5-hmC at 4°C with rotation. 3μg of DNA was then mixed with TE buffer and sonicated (200–800 bp) for 20 minutes at 20% amplitude on the Q800R2 DNA sonicator; samples were run on a gel to verify size. DNA was then denatured at 95°C for 15 minutes and immediately cooled on ice. Two thirds of the sample was combined with an IP dilution buffer (1M Tris, 10% SDS, Triton X-100, 0.5M EDTA, NaCl) and the other third kept for Input fraction. Diluted DNA was then added to antibody-bead complexes and incubated overnight at 4°C with rotation. Immune complexes were then sequentially washed with low salt buffer, high salt buffer, LiCl immune complex buffer, and twice with TE buffer. After proteinase K digestion (TE buffer, 1% SDS, proteinase K 10 ul/mL) at 55°C for 2 hours at 800 RPM, DNA was eluted at 95°C for 10 minutes at 800 RPM. DNA was subsequently extracted by phenol/chloroform/isoamyl alcohol and then precipitated in isopropanol. Immunoprecipitated DNA was then subjected to quantitative real-time PCR using primers specific to CpG islands within the promoter region of *Pomc* (F: AAGGTGTACCCCAATGTCGC; R: TTCTCGGTATCCGGCTCCA). The cumulative fluorescence for each amplicon was then normalized to input amplification.

### Direct bisulfite sequencing

Genomic DNA (50 ng) was bisulfite converted through the Qiagen EpiTect Bisulfite Kit and amplified using a nested PCR protocol in which 0.4 μl of the forward and 0.4 μl of the reverse primers (5 μМ) were mixed with 1 μl of bisulfite treated DNA, 3.2 μl of nuclease free water, and 5 μl of HotStarTaq Master Mix (#203443, Qiagen). Samples were run on a thermal cycler for target amplification using the methyl-specific primers for *Pomc* (F: AAGTTTTTGTTTAGTTTTGAGTGGAG; R: TCTTCTCTCTTCTTTTATACCTACAAAATT). PCR parameters were as follows: 95°C for 5 min, then 49 cycles of 95°C for 1 min, 60°C for 1 min and 72°C for 1 min, followed by a 5 min incubation step at 72°C. Then, 1 μl of the product from the first set of primers was used to repeat the experiment with the same parameters and mix using methyl-specific primers with a product nested inside that of the first primers (F: TTTTTAATTAAGTTTTTTTTGATTAT; R: CTTCTCTCTTCTTTTATACCTACAAAATTA). Product sizes were verified on a gel. PCR product was cleaned using ExoSAP-IT (Affymetrix, Santa Clara, CA) and sequenced by the Genomics Sequencing Center at the Fralin Life Sciences Institute of Virginia Tech using the first reverse primer. Analysis was done using Chromas software to read the electropherogram and percent methylation of the CpG sites was determined by the ratio between peak values of guanine (G) and cytosine (C), C/(C + T) * 100.

### Chromatin immunoprecipitation

One hemisphere of hypothalamus tissue was fixed in PBS with 1% formaldehyde for 10 min at 37°C and then glycine (2.5 M) was added to quench the reaction. Fixed tissue was then extensively washed in PBS before being homogenized in a hypotonic buffer (10 mM KCl, 20 mM HEPES, 1 mM MgCl, 1 mM DTT) with protease inhibitors. Homogenates were centrifuged at 1350 x g for 10 min at 4°C and the resulting pellets (nuclei) were resuspended, sheared and immunoprecipitated with the Epigentek Cut & Run kit (#P-2028-24) according to the manufacturer’s instructions. We then used Phenol/chloroform/isoamyl alcohol and then ethanol- precipitation to isolate DNA, which was used quantitative real-time PCR with the same primers specific to the rat *Pomc* promoter described above for meDIP. For analysis, the cumulative fluorescence for each amplicon was taken as a percentage of the input fraction and taken as a fold change of the control group.

### Statistical analyses

All data are presented as mean with standard error. Data with two group comparisons were analyzed using unpaired two-tailed *t*-tests. Time-course weight data was analyzed with two-way ANOVA and Fisher LSD posthoc tests. Statistical outliers were defined as those samples that were two or more standard deviations from the mean and were determined by the outlier function in Prism (Graphpad).

## Results

### Exposure to a high fat diet increases *Pomc* methylation and reduces gene expression

**Fig 1:**
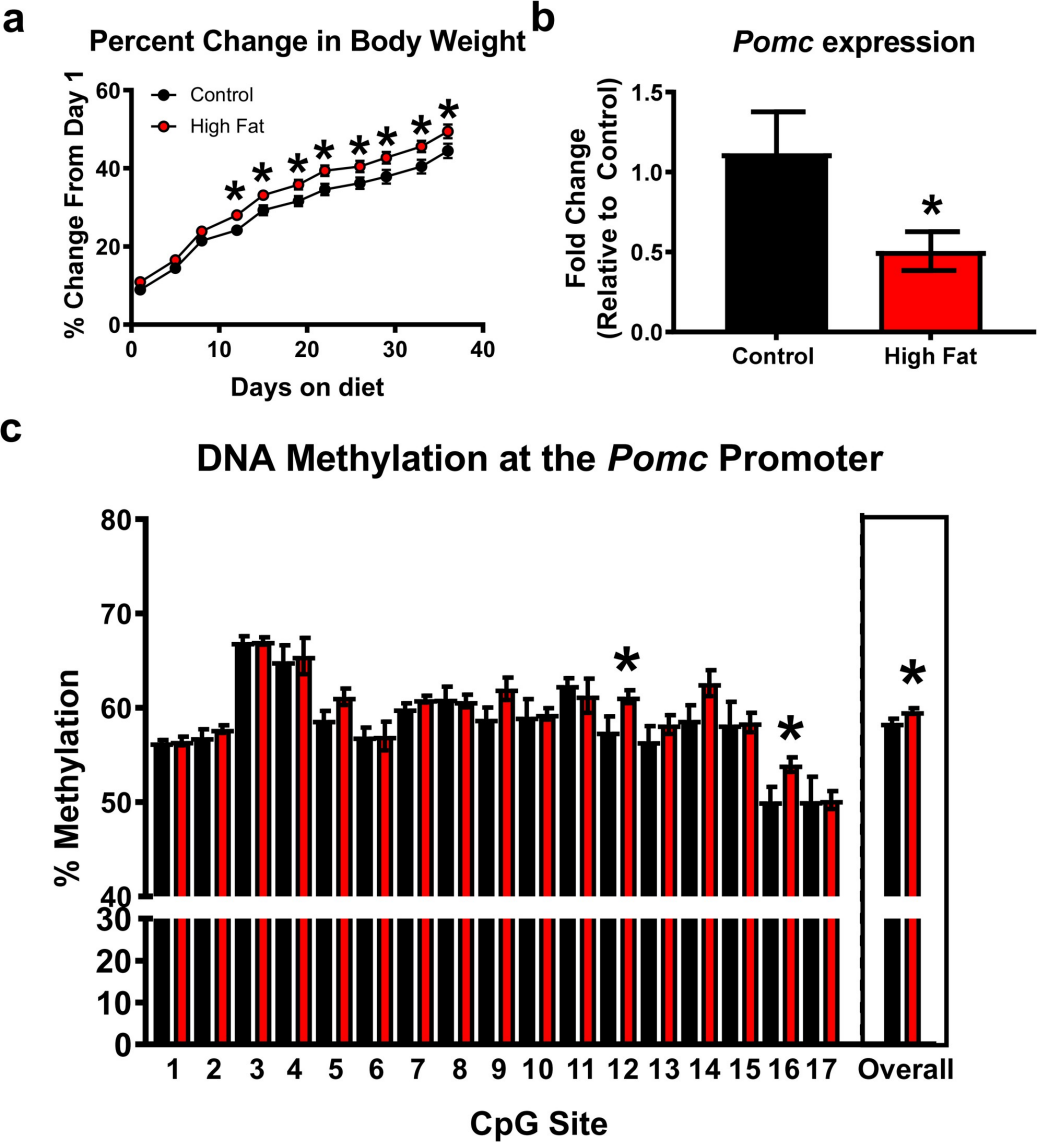
Exposure to a high fat diet increases *Pomc* methylation and reduces gene expression in the hypothalamus. Young adult male rats were started on a high fat diet (n = 10) or standard rat chow (Control; n = 10) and remained on this diet for a total of 7 weeks. (**a**) Percentage change in body weight over time was greater in the high fat diet fed animals relative to controls. (**b**) Quantitative real-time PCR revealed that animals fed the high fat diet have reduced expression of *Pomc* compared to controls (n = 8–9 per group). (**c**) There was a significant increase in overall methylation at the promoter region of *Pomc* of high fat fed animals compared to controls (boxed area; n = 7–10 per group). *P < 0.05 from Control.

To begin examining altered methylation at *Pomc*, we first established that our high fat diet paradigm would result in abnormal weight gain over the course of 6 weeks relative to a standard rat chow diet. For six weeks the animals were fed one of two diets, standard rat chow (22% protein, 4% fat, 5% fiber) or a high fat diet (60% fat). As the groups differed in initial body weight, we examined percentage change in body weight from their baseline to determine the effectiveness of the high fat diet. We found from day 1 to day 42 there was a significant difference between groups with the high fat diet gaining greater percentage of weight over time relative to controls (Group: F_(1,18)_ = 5.703, P = 0.0281; Time: F_(10,180)_ = 747.6, P < 0.0001; Interaction: F_(10,180)_ = 1.913, P = 0.0459; Fig 1A). Thus, by the end of the 6-week diet the high fat fed animals had a significantly higher increase in body weight than did controls.

Next, we evaluated the potential relationship between the observed increase in weight gain and *Pomc* methylation and gene expression. To analyze *Pomc* RNA levels, we used quantitative real-time PCR and found that animals fed the high fat diet have reduced expression *Pomc* compared to controls (t_(15)_ = 2.046, P = 0.0587; Fig 1B). Next, we analyzed DNA methylation levels at the gene promoter of *Pomc* using bisulfite sequencing to determine if the changes in gene expression were related to altered DNA methylation patterns. We found an overall significant increase in methylation of high fat fed animals compared to controls (t_(15)_ = 2.445, P = 0.0273; Fig 1C), as well as individual increases at CpG12 and CpG16. However, the overall change suggests broad increases in DNA methylation of the *Pomc* promoter as a result of the high fat diet. Collectively these results support prior work [14] and suggest that the *Pomc* gene becomes repressively methylated following prolonged exposure to a high fat diet.

### CRISPR-dCas9-Tet1 demethylation of *Pomc* does not prevent abnormal weight gain

**Fig 2:**
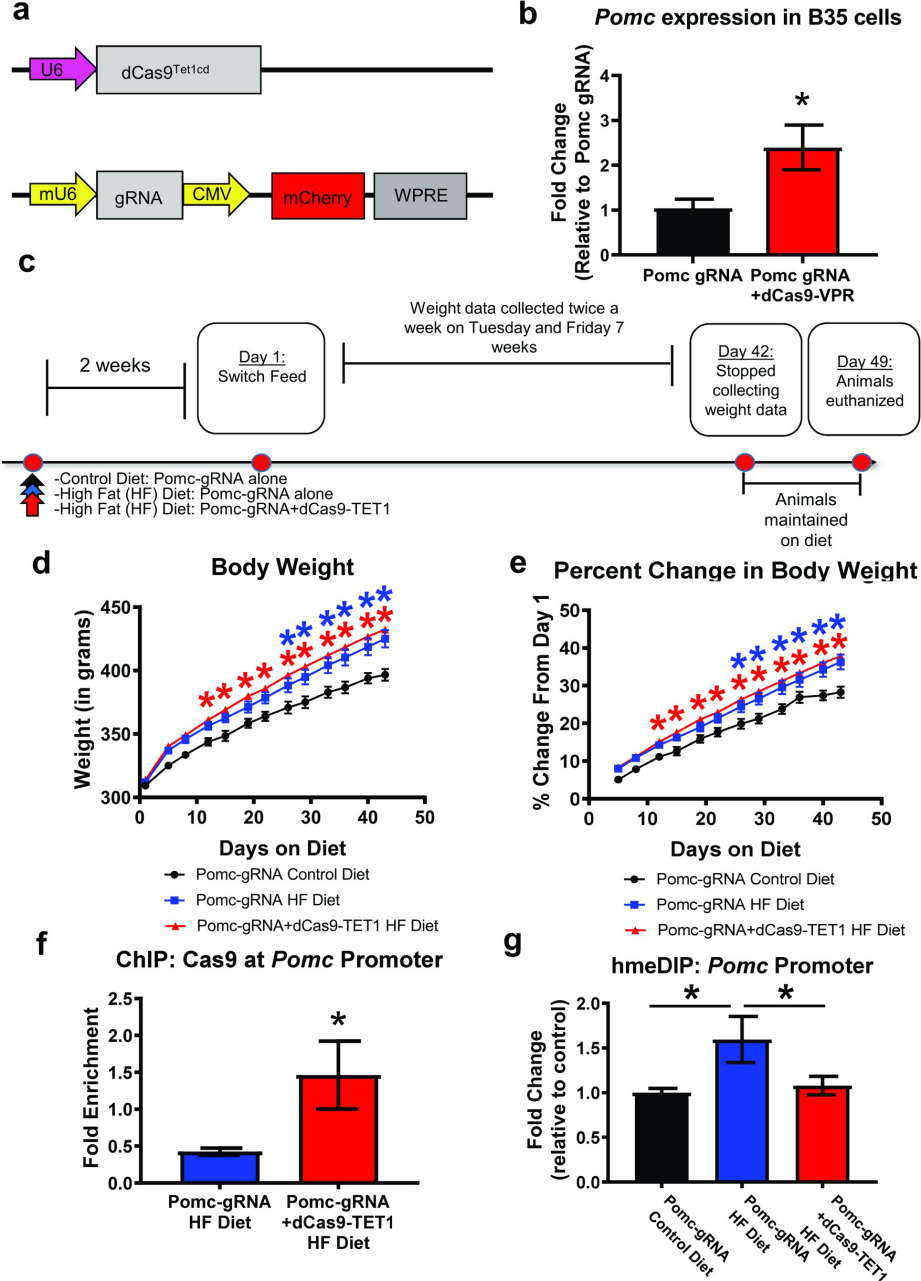
CRISPR-dCas9-TET1 demethylation of *Pomc* does not prevent abnormal weight gain on a high fat diet. (**a**) Schematic showing the modified CRISPR-dCas9 system where TET1 is fused to a catalytically inactive Cas9. (**b**) In rat B35 cell culture there was a significant increase in *Pomc* expression when *Pomc*-specific guide RNA (gRNA) was combined with the transcriptional activator dCas9-VPR (n = 3 per group). (**c**) The *Pomc*-specific gRNA developed in B and dCas9-TET1 plasmids were infused into the hypothalamus. Two weeks post infusion, animals were placed on a control or high fat diet and maintained for 7 weeks. Weight data was collected during the first 6 weeks and whole hypothalamus tissue collected at the end of the 7^th^ week. (**d-e**) No significant changes were observed in body weight or percent change in body weight over time in the gRNA+dCas9-TET1 group relative to high fat fed controls. However, *Pomc*-gRNA+dCas9-TET1 resulted in faster weight gain relative to standard diet control diet in comparison with gRNA only high fat diet fed controls (n = 8 per group). (**f**) ChIP analysis revealed an increase in Cas9 enrichment at the *Pomc* promoter within animals infused with the dCas9-TET1 plasmid relative to similar fed control group, confirming that our dCas-TET1 complex was correctly targeted to the *Pomc* promoter. (**g**) Gene-specific (*Pomc*) 5-hmC methylated DNA immunoprecipitation (meDIP) analysis revealed significant increases in 5-hmC levels within the animals injected with only the gRNA and fed a high fat diet, which was lost in gRNA+dCas9-TET1 injected high fat fed animal. *P < 0.05 from Control.

To determine if promoting the 5-hmC state of the *Pomc* promoter would prevent abnormal weight gain on a high fat diet, we used a modified version of the CRISPR-dCas9 system in which TET1 is fused to a catalytically inactive Cas9 (Fig 2A). We developed a guide RNA (gRNA) sequence against the *Pomc* promoter and found that in rat B35 cell culture there was a significant increase in *Pomc* expression when combined with the transcriptional activator dCas9-VPR (t_(4)_ = 2.514, P = 0.0329; Fig 2B), indicating that our gRNA was correctly targeting the *Pomc* promoter. Next, we used this gRNA *in vivo* with or without the dCas9-TET1 plasmid (Fig 2C). These plasmids were infused into the hypothalamus prior to obesity-development. Two weeks post infusion, a time point at which we have observed strong expression of CRISPR plasmids in the hypothalamus [22], animals were placed on a control or high fat diet and maintained on this for 7 weeks, over which we collected weight data during the first 6 weeks. Surprisingly, though we did observe significant effects for Group (F_(2,21)_ = 4.504, P = 0.0236), Time (F_(12,252)_ = 735.8, P < 0.0001) and a Group x Time Interaction (F_(24,252)_ = 4.975, P < 0.0001; Fig 2D), we did not observe any significant changes in body weight from the dCas9-TET1 in high fat animals relative to control injected high fat animals. Similarily, though for percentage change in bodyweight we did observe significant effects for Group (F_(2,21)_ = 7.004, P = 0.0047), Time (F_(11,231)_ = 593.8, P < 0.0001) and a Group x Time Interaction (F_(22,231)_ = 2.935, P < 0.0001; Fig 2E), we again did not observe an effect of the dCas9-TET1 plasmid on the percentage change in body weight over time in high fat fed relative to control injected high fat animals. However, on average the dCas9-TET1 manipulation was enhancing the speed of weight gain as they differed from control diet animals sooner than did control injected high fat diet animals. To confirm our manipulation was effective, we performed ChIP analysis on hypothalamus tissue from high fat fed animals and found an increase in dCas9 enrichment at the *Pomc* promoter within animals infused with the dCas9-TET1 plasmid relative to control (t_(14)_ = 2.247, P = 0.0413; Fig 2F), confirming that our dCas9-TET1 complex was correctly targeted to the *Pomc* promoter. Interestingly, gene-specific (*Pomc*) 5-hmC meDIP analysis revealed significant increases in 5-hmC levels within the animals injected with only the gRNA and fed a high fat diet, which was not present in high fat animals receiving the gRNA with dCas9-TET1 (F_(2,20)_ = 3.671, P = 0.0439; Fig 2G); this meDIP approach was used because bisulfite sequencing cannot distinguish between different forms of DNA methylation, such as 5-mC from 5-hmC, and due to our observation of broad increases in methylation across the *Pomc* promoter in our initial experiment (Fig 1). Though this demethylation further confirms the correct targeting of *Pomc* promoter with the dCas9-TET1 plasmid, it surprisingly suggests that *Pomc* repression may be related to DNA 5-hmC, an epigenetic mark typically associated with transcriptional activation though can also lead to gene repression [24]. However, this could also be an indication that active demethylation may have started to occur at the *Pomc* promoter to account for the high fat diet-induced reductions in *Pomc* expression, which was accelerated by our dCas9-TET1 manipulation. Regardless, our dCas9-TET1 manipulation resulted in demethylation of *Pomc* but was insufficient to slow abnormal weight gain on the high fat diet. Together, these data suggest that targeted *Pomc* promoter demethylation is not sufficient to prevent abnormal weight gain during exposure to a high fat diet.

### CRISPR-dCas9-DNMT3a methylation of *Pomc* does not influence weight gain

**Fig 3:**
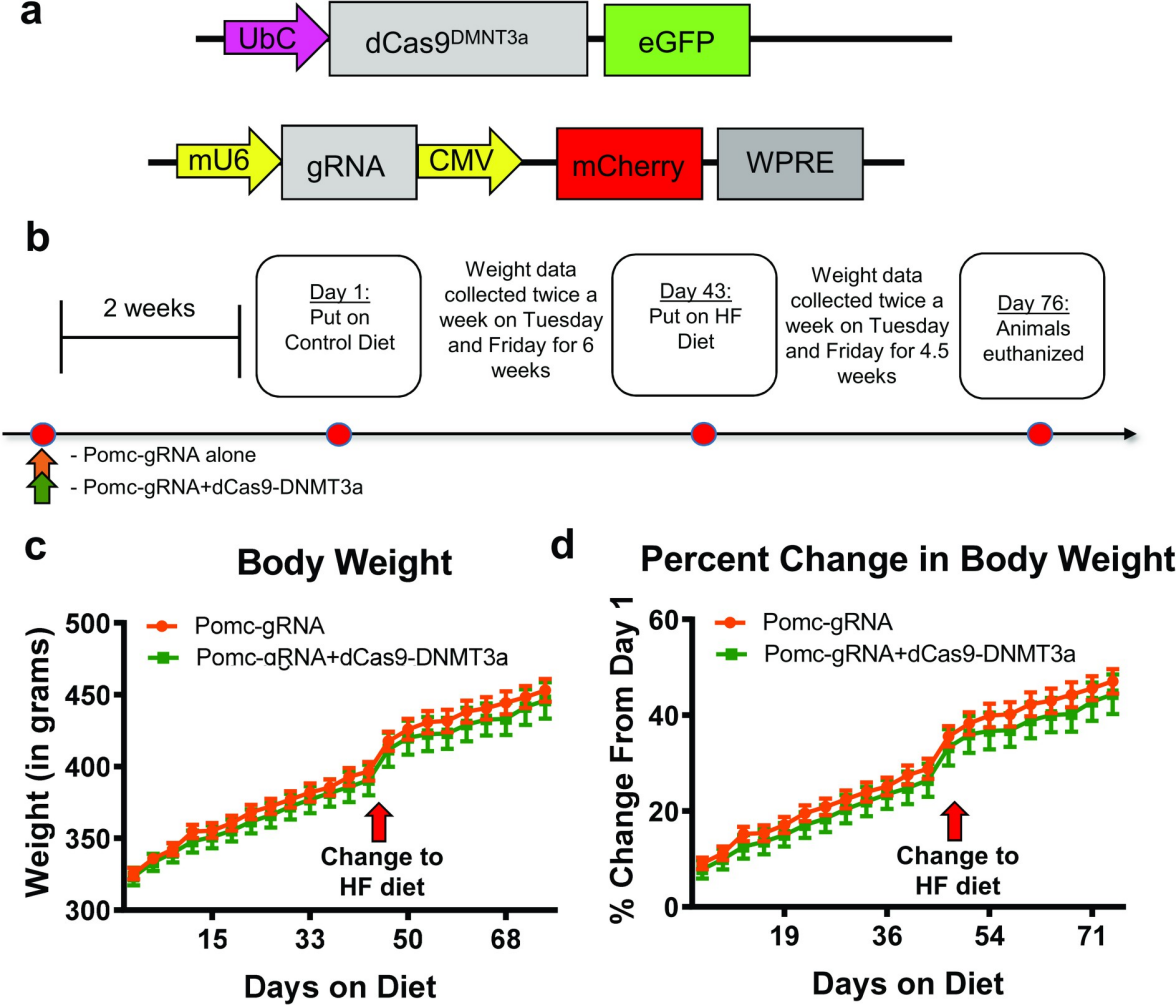
CRISPR-dCas9-DNMT3a methylation of *Pomc* does not lead to abnormal weight gain on standard or high fat diets. (**a**) Schematic showing the modified CRISPR-dCas9 system where DNMT3a is fused to a catalytically inactive Cas9. (**b**) The gRNA developed in Fig 2 and dCas9-DNMT3a plasmids were infused into the hypothalamus. Two weeks post infusion, animals were placed on a control diet and maintained for 6 weeks. After week 6, all animals were switched to a high fat diet for the next 4.5 weeks. Body weight data were collected every 3–4 days throughout the entire diet procedure. (**c-d**) CRISPR-dCas9-DNMT3a targeting of *Pomc* did not significantly change body weight (**c**) or percent change in body weight (**d**) over the diet procedure (n = 8–12 per group).

To confirm our unexpected result using the dCas9-TET1 system, we next tested if repressively methylating *Pomc* in the hypothalamus could control weight gain. In order to do this, we used our *Pomc* gRNA in combination with a dCas9-DNMT3a plasmid (Fig 3A). Similar to the previous experiment, we used the gRNA *in vivo* with or without the dCas9-DNMT3a plasmid (Fig 3B) and these plasmids were injected into the hypothalamus prior to weight being monitored. Two weeks post infusion, animals were maintained on a control diet for 6 weeks and weight data was collected twice per week. We then switched all the animals to a high fat diet throughout the last 4.5 weeks, providing a within-subjects design to monitor changes in bodyweight from our dCas9-DNMT3a targeting of the *Pomc* promoter in both control and high fat diets. Surprisingly, we did not observe any significant changes in body weight over the course of 10.5 weeks from the dCas9-DNMT3a manipulation (Group: F_(1,17)_ = 0.3241, P = 0.5766; Time: F_(21,357)_ = 487, P < 0.0001; Interaction: F_(21,357)_ = 0.3891, P = 0.9938; Fig 3C), nor did we observe any significant changes in percentage change in body weight over time between either group (Group: F_(1,17)_ = 0.4804, P = 0.4976; Time: F_(20,340)_ = 473.4, P < 0.0001; Interaction: F_(20,340)_ = 0.3992, P = 0.9912; Fig 3D). These data suggest that using the CRISPR-dCas9-DNMT3a to repressively methylate the *Pomc* promoter is not adequate to mimic abnormal weight gain. In combination with our prior data, these results suggest that changes in the methylation state of *Pomc* are a consequence of, but do not contribute to, abnormal weight gain during the development of obesity.

## Discussion

Obesity is a common risk factor for numerous health conditions and POMC deficiency has been shown to be associated with the development of this disease [15–18]. Previous work had showed that exposure to a high fat diet was assoicated with increased DNA methylation at the *Pomc* promoter region, leading to a decrease in *Pomc* expression in the hypothalamus [14]. However, it was unknown if DNA methylation-mediated repression of *Pomc* in the hypothalamus contributed to, or was a consequence of, abnormal weight gain during the development of obesity. Here, we found that CRISPR-dCas9-TET1-mediated demethylation of *Pomc* was not sufficient to prevent abnormal weight gain following exposure to a high fat diet. Additionally, in an attempt to mimic obesity development we found that CRISPR-dCas9-DNMT3a-mediated methylation of *Pomc* was not sufficient to alter weight gain following exposure to control or high fat diets. Together, these results suggest that altered *Pomc* DNA methylation levels likely do not directly contribute to the development of the obesity phenotype.

While previous studies have reported altered methylation levels at the *Pomc* promoter following exposure to a high fat diet [14], none had directly manipulated the methylation state of this gene. Here, we confirm the previously reported increase in repressive methylation of *Pomc* and provide the first evidence that CRISPR-dCas9-mediated *Pomc* demethylation does not prevent abnormal weight gain following exposure to an obesogenic diet. These data imply that the dysregulation of this major satiety gene is not the target mechanism for obesity development. Overall, these data indicate that the altered methylation state of the *Pomc* promoter is a consequence of exposure to a high fat diet rather than the cause of significant weight gain and obesity development. However, this raises questions regarding how *Pomc* loss contributes to abnormal weight gain as prior work has shown that *Pomc* deficiency is associated with the development and progression of obesity [15–18]. Considering that our data indicate repression of *Pomc* by DNA methylation, it is surprising then that demethylating this gene did not slow the progression of weight gain. It is important to note though that prior work has looked at *Pomc* loss, where the genomic sequence on at least one allele is removed from the genome. In contrast, in our study the genomic information remained present but the accessibility of this DNA region was altered via DNA methylation, resulting in a very different cellular state than the prior work. Furthermore, the prior studies have observed increased susceptibility to obesity when *Pomc* is lost, though ours tried to prevent normal progression of DIO-mediated weight gain via controlling the endogenous expression of *Pomc* via epigenetic modifications. Thus, our work takes a very different approach from that of prior studies, which is what could have resulted in the divergent outcomes. Regardless, it is of critical importance in future studies to better determine how controlling the DIO-mediated changes in *Pomc* epigenetic regulation can contribute to the development and progression of obesity.

As with the majority of studies, the design of our current study is subject to some limitations. The first is that our diet was short-term, lasting only 7 weeks. Important here is that the existing literature is inconsistent when it comes to approaches used in obesogenic animal models as there have been varied reports on the type of diet, age of animals, and duration of feeding used in DIO work [9, 14, 25–27]. Although we can’t say for certain that the animals are obese due to the short time frame used, they were gaining significantly more weight than controls over this period of time. Thus, we are confident that duration of feeding is not a major limiting factor in interpreting our results. Second, our animals were group housed and while the same diet was given to a specific cage, the animals within this often received different CRISPR-dCas9 manipulations. As a result, we were unable to accurately track and calculate individual food intake following our CRISPR-dCas9 manipulations. This is important as it is possible the CRISPR-dCas9-TET1 manipulation of *Pomc* may have altered food intake independent of changes of weigh gain. Perhaps this could account for why the animals receiving the dCas9-TET1 manipulation tended to show faster weight gain on the high fat diet than did controls. However, this would have been paradoxical considering that POMC increases should lead to satiety though increases in *Pomc* expression from our CRISPR-dCas9 manipulation could have indirectly altered expression of other satiety genes, such as neuropeptide Y (NPY), which could lead to greater food intake and weight gain. It will be interesting in future studies to test then if CRISPR-dCas9-TET1 manipulation of *Pomc* leads to changes in food intake. Also, we did not test the conditions under which CRISPR-mediated regulation of *Pomc* alters weight gain in female rats, leaving questions about whether *Pomc* methylation could contribute to obesity development in a sex-specific manner. Additionally, our CRISPR manipulations were cell-type independent, persistent and occurred prior to animals being placed on the diet. This raises important questions about the cell-type specificity of hypothalamic gene regulation during obesity development, as well as whether a more temporally controlled approach may have resulted in a different outcome, though no such approach exists, to date. Further, as we were unable to quantify body fat percentage, it is possible that the dCas9-TET1 manipulation of the *Pomc* promoter was able to slow increases in body fat accumulation, even if weight gain was not changed. However, body fat percentages generally take more time to increase on obesogenic diets than the timeline used here, making body weight the ideal variable to evaluate from our manipulation. Despite these limitations, our data provide the first evidence that controlling the DNA methylation state of *Pomc* in the hypothalamus is likely not sufficient to prevent abnormal weight gain during the development of obesity.

## Conclusion

In conclusion, we found that CRISPR-dCas9 mediated demethylation of the *Pomc* promoter was insufficient to prevent abnormal weight gain following acute exposure to an obesogenic diet. Further, promoting a repressive epigenetic state at the *Pomc* promoter was also insufficient to produce abnormal weight gain on regular or high fat diets. These data advance our understanding of how diet-induced epigenetic regulation of *Pomc* in the hypothalamus contributes to the development and progression of obesity, which could have important therapeutic implications for the treatment of this major disease.

## Supporting information

S1 File Original data. Original data used to make graphs presented in Figs 1–3.
(XLSX)
